# Multi-center randomized double-blind controlled clinical study of chemotherapy combined with or without traditional Chinese medicine on quality of life of postoperative non-small cell lung cancer patients

**DOI:** 10.1186/1472-6882-12-112

**Published:** 2012-08-01

**Authors:** Ling Xu, HeGen Li, ZhenYe Xu, ZhongQi Wang, LingShuang Liu, JianHui Tian, JianLi Sun, Lei Zhou, YiLin Yao, LiJing Jiao, Wan Su, HuiRu Guo, PeiQi Chen, JiaXiang Liu

**Affiliations:** 1 Department of Oncology, Longhua Hospital, Shanghai University of TCM, 725 Wanping road, Shanghai 200032, China

## Abstract

**Background:**

Traditional Chinese medicine (TCM) is a widely applied complementary therapy for cancer patients. It can reduce the chemical drugs induced toxic effects to improve the quality of life (QOL). This study applies the highest quality of clinical trial methodology to examine the role of TCM in improving QOL of postoperative non-small-cell lung cancer patients.

**Methods and design:**

This study is a multi-center, randomized, placebo-controlled, double-blind trial. Four hundred eighty patients will be recruited into seven different research centers in China. These patients that meet the inclusion criteria will be randomized into either a treatment group or a placebo group. Each group will receive treatments of 3-weekly chemotherapy with TCM or placebo for four cycles. The primary outcome will involve the evaluation of QOL and the secondary outcome assessments will include two-year disease-free survival rate and disease-free survival. Other efficacy assessments are changes of TCM symptoms and toxicity. Side effects and safety profile of the therapy would be evaluated at the same time. The investigators expect that TCM therapy combined with chemotherapy is superior to chemotherapy solely in terms of QOL improvement and disease-free survival extension. "Intention-to-treat" analysis will include all randomized participants.

**Discussion:**

The results from the clinical trial will provide evidence for the effectiveness of chemotherapy combined with or without TCM in QOL of postoperative NSCLC patients.

**Trial registration:**

Clinical Trials.gov (Identifier: NCT01441752).

## Background

Lung cancer is one of the most common malignant tumors in the world, accounting for 26–29% of all cancer deaths [1]. Increasingly, lung cancer is becoming a global health problem, with greater than 1 million deaths annually attributed to lung cancer [2]. Non-small cell lung cancer (NSCLC) accounts for approximately 80% of all lung cancer diagnoses and more than 75% of the patients are diagnosed at an advanced stage. Consequently, the prognosis for NSCLC continues to remain poor, with a 5-year survival of 15% [3].

At present, the high rate of recurrence and metastasis of postoperative non-small cell lung cancer (NSCLC) patients is one of the leading causes resulting in failure of treating lung cancer. More than 35% of postoperative lung cancer patients with stage I died in 5 years due to recurrence or metastasis; the 5-year survival rate of stage II, IIIa, IIIb was 31%, 17.9% and 11.7% respectively. The survival rate was improved by 5% with adjuvant chemotherapy after resection, so regimen consist of platinum-based two chemical medicines are commended as the adjuvant chemotherapy for treating postoperative NSCLC patients, but the toxicity and side effects of chemotherapy such as nausea and vomiting, hair loss, and bone-marrow depression can decrease quality of life (QOL) of patients [4-6]. These adverse reactions need to be treated, otherwise which will impact the completion of treatment. Literature and preliminary studies have shown that traditional Chinese medicine (TCM) can prolong survival and improve QOL, but high-level evidence is desperately needed to support this finding [7].

Over the past 10 years, many oncologists focused on survival advantage of adjuvant chemotherapy of postoperative non-small cell lung cancer patients. Data from JBR.10 study showed that overall QOL and functional domain scores are somewhat compromised after thoracotomy and pneumonectomy; but scores improve fairly early in patients who are not receiving chemotherapy [8]. As expected, adjuvant chemotherapy has an immediate negative impact on a number of aspects of QOL in individuals with NSCLC who have undergone resection with curative intent [9]. Chief among these is the symptoms that reflect most of the serious adverse effects associated with platinum-based chemotherapy: fatigue, loss of appetite, nausea, and vomiting. Therefore, QOL in clinical assessment has become more and more prominent in clinical research [10].

Over the past decade, Chinese medicine has made progress in the treatment of lung cancer patients. Chinese medicine has some unique advantages over chemical drugs, such as improving the quality of life, reducing recurrence rate and prolonging overall survival, etc. Regarding KPS score, traditional Chinese medicine can stabilize and improve the KPS scores after surgery in 82.0% to 90% patients [11,12]. In addition, it can improve QOL significantly by using NCI-L evaluation. 85% to 87.5% of postoperative patients can stabilize and improve the body weight after receiving TCM therapy [11-15]. Two comparative studies from Japan indicated Chinese herbal medicine to be effective in reduction of chemotherapy-induced muscle pain and diarrhea [16,17].

Professor Liu Jiaxiang in Longhua hospital affiliated to Shanghai university of TCM has been doing clinical research on advanced lung cancer patients since the 1960s. His team have completed four prospective randomized clinical studies supported by China's State Science and Technology. The result showed that disease stable rate was 67.83%, 1, 3 and 5-year survival rate was 60.94%, 31.86% and 24.22% respectively in the treatment group. Median survival was 417 days (13.9 months). The immune function and QOL were improved after treated with combined Chinese and Western medicine compared to the control group [18-25]. This reflected characteristics and therapeutic effect of Chinese medicine on treatment of lung cancer based on syndrome differentiation. But these studies have methodological issues and can’t be widely accepted worldwide.

This paper has been compiled to outline the methodology for a randomized-controlled clinical study the effect of chemotherapy combined with or without TCM in QOL of postoperative NSCLC patients that adhere to the CONSORT [26] checklist in its design.

### Study aims

The primary aim of this study is to evaluate the effectiveness of chemotherapy combined with or without TCM in QOL of postoperative NSCLC patients. The secondary aims are to compare the disease-free survival time (DFS), two-year disease-free survival rate and safety of intervention in the two groups.

## Methods

### Study design

**Figure 1:**
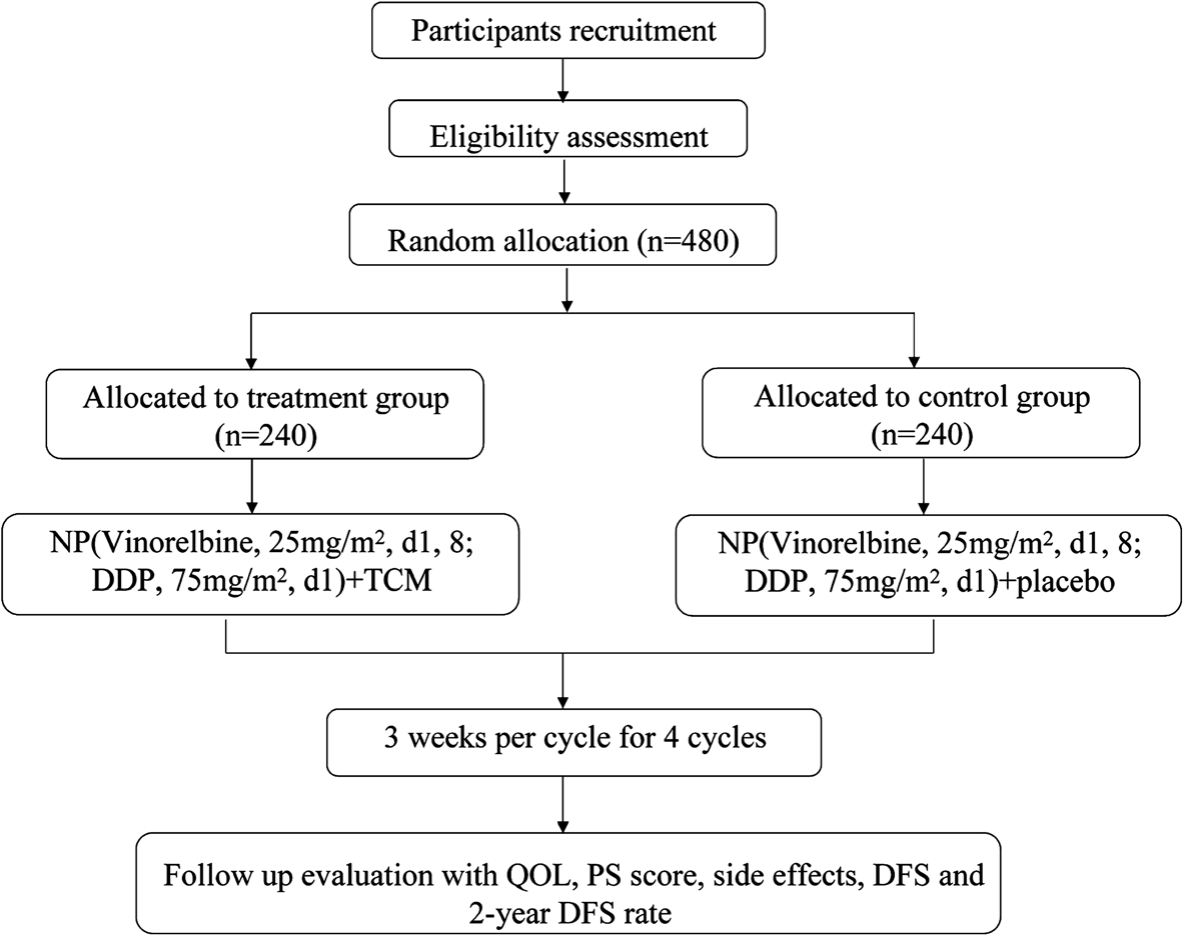
Flow diagram of the study.

This study is a multi-centered, randomized, placebo-controlled, double-blind trial. Subjects from seven clinical research centers in China will engage into the trial. Longhua hospital affiliated to Shanghai university of TCM, Shanghai Chest Hospital affiliated to Shanghai Jiao Tong University, Ruijin Hospital affiliated to Shanghai Jiao Tong University, Huadong hospital affiliated to Fudan university, NO.6 people’s hospital affiliated to Shanghai Jiaotong university and Changzheng Hospital of the Second Military Medical University. Eligible participants will be randomly allocated into either of the two groups (chemotherapy with TCM or chemotherapy with placebo) and will receive treatment for 4 cycles (Figure 1).

### Randomization

Dynamic stratified and randomized method is applied in this study. Random numbers will be automatically generated by computer referring to pre-configured stratified factors (stages, pathological types, ages and TCM syndromes). Shanghai Clinical Research Center will provide the random number to each participating center. The block size and treatment-assignment table will not be available to the researchers until the end of the study.

### Target population and selection

NSCLC patients received complete resection with confirmed pathological diagnosis of stage Ib-IIIa; TCM syndromes are Yin deficiency, Qi deficiency, Qi and Yin deficiency, Spleen and Kidney deficiency.

### Diagnostic criteria

Diagnostic criteria for primary bronchial lung cancer: According to the "New compiled diagnosis and treatment norms of common malignant tumors" (Volume IX - primary bronchial lung cancer, the Chinese Anti-Cancer Association compiled, Beijing: Chinese Union Medical University Press, 1997: 737–781).

TNM stage of primary bronchial lung cancer: According to the staging system of 2009 International Union Against Cancer (UICC), version 7.

### Syndrome differentiation criteria

Syndrome differentiation criteria based on "The Guiding Principles of Clinical Research of New Chinese Medicine (trial)" (China Pharmaceutical Technology Publishing House, 2002) and "Shanghai diagnosis and treatment routine of TCM Syndrome"(Shanghai Municipal Health Bureau edit). Four syndromes of TCM are set as follows:

Qi deficiency syndrome is composed of the following symptoms: coughing, phlegm, poor appetite, spiritlessness and weakness, light and plump tongue; secondary symptoms of spontaneous sweating, loose stool or soft slippery pulse.

Yin deficiency syndrome contains main symptoms of coughing, less phlegm, dry mouth, red tongue; secondary symptoms of the night sweating, heartburn and insomnia, low fever, thread and rapid pulse.

Qi and Yin deficiency syndrome contains main symptoms of coughing, less phlegm, shortness of breath, spiritlessness and weakness, thirst without the desire to drink; secondary symptoms of spontaneous sweating, night sweating, reddish tongue or tongue with teeth marks, and thread and weak pulse.

Spleen and Kidney deficiency syndrome contains main symptoms of coughing, shortness of breath, dyspnea after movements, weakly coughing; secondary symptoms of soreness of loins, tinnitus, loose stool, spiritlessness and weakness, light tongue or tongue with teeth marks, and deep thready and weak pulse.

The diagnosis can be given with the conditions of at least two of the main symptoms and one of the secondary symptoms.

### Inclusion criteria

Meet the diagnostic criteria of primary bronchial lung cancer, and pathologically or cytologically confirmed of squamouscarcinoma, adenocarcinoma, adenosquamous carcinoma or large cell carcinoma of the lung, age between 18 and 75 years old; TCM syndromes are Yin deficiency, Qi deficiency, Qi and Yin deficiency or Spleen and Kidney deficiency, Eastern Cooperative Oncology Group (ECOG) performance status of two or less, Stage Ib ~ IIIa with complete resection, first chemotherapy treatment within 6 weeks after operation, normal hematological function with total neutrophil count >1.5 × 10^9^/l and platelets >80 × 10^9^/l, normal liver function and kidney function, voluntarily involved to clinical study and sign informed consent.

### Exclusion criteria

Suffering from other primary malignant tumors; incomplete resection or uncertain to take resection; serious problem of heart, liver or kidney with severe dysfunction; being pregnant or breast-feeding, mental or cognitive disorders that would influence judgment of QOL in this study; during the period of adjuvant chemotherapy, or prior chemotherapy; being participating other drug trials; allergy to the drug in the study. Any patient with problems mentioned above will be excluded from this clinical study.

### Sample size

Refer to the results of JRB.10 study, for early lung cancer patients after three-month adjuvant chemotherapy, the QOL score assessed by QLQ-C30 scale decreased 27% compared with baseline. The sample size of this study is based on the validity of assumptions and clinical experience of the past. Compared to the baseline, it is estimated that the QOL score assessed by QLQ-C30 scale for patients treated with three-month postoperative adjuvant chemotherapy, there was 15% of patients with no deterioration of QOL combined with TCM than treatment without TCM. Inspection level α = 0.05, grasp 1-beta = 0.90, compared with the baseline, the QOL score assessed by QLQ-C30 scale after three-months in the treatment group decline 12% while 27% in the control group, made 198 cases of sample size in each group and consider 20% off rate, it is expected that the research team observed 480 cases (n = 240 cases) of patients in 2 years to compare QOL of postoperative NSCLC patients by chemotherapy with or without TCM.

### Intervention

Researchers will undertake a one-day training course for this trial. This course will cover the study protocol, the recording method for the clinical record form (CRF), and basic information about clinical research, a researcher's responsibility and research monitoring.

### Chemotherapy

The chemotherapy for NSCLC patients is a combination of Vinorelbine, 25 mg/m^2^, d1, 8 and DDP, 75 mg/m^2^, d1 (NP) giving three-weekly for four cycles. For patients who receive fewer than the intended number of cycles of chemotherapy, the study duration will be calculated by the basis of the projected interval.

### Chinese herbal medicine

Prescriptions formulated into granules origin from Professor Liu Jiaxiang in Longhua hospital. Package of granules is made into three types with functions such as benefiting Qi recipe, benefiting Yin recipe and detoxication and resolving masses recipe (Table 1). Each package contained 20 g of water-soluble herbal granules that were manufactured at a Good Manufacture Practice standard facility (Tian Jiang Ltd, Jiangyin, China). Each package was labeled with a serial number. The prescription form comprised the stock list with both the name and serial number.

**Table 1:** Dosage of Chinese medicine according to different syndromes

**Syndrome Differentiation**	**Chinese Medicine (unit:package)**		
	**Benefiting Qi Recipe**	**Benefiting Yin Recipe**	**Detoxication and Resolving Masses Recipe**
Qi Deficiency	1	—	2
Yin Deficiency	—	2	2
Qi and Yin Deficiency	1	1	2
Spleen and Kidney Deficiency	2	—	2

### Placebo

It is difficult to define a pure placebo in Chinese herbal medicine where all natural substances are potentially therapeutic. So we compromise the raw materials for the placebo including 10% of Chinese medicine, food color and artificial flavors. The placebo and therapeutic packages were stored in different cabinets, and only the dispensing technician knew the contents of the packages.

### Primary outcome measurement

#### Quality of life

Quality of life scale: QLQ-LC43 scales made by EORTC will be applied to assess the quality of life of patients. Scoring method is used to assess the results according to the score changes [27]. Scores of general QOL and each field is calculated respectively by international uniform scoring system prior and after intervention. Performance status of patients physical conditions are assessed according to ECOG PS standard before and after each treatment.

### Secondary outcome assessments

Two-year disease-free survival rate: the patients without recurrence and metastasis within 2 years after resection account for percentage of all patients.

Disease-free survival (DFS) :it refers to the interval time either from the first date of medication to that of recurrence and metastasis observed, or death for any reason (record according to the first event). The date of last evaluation will be recorded if patients either have no recurrence and metastasis when reach censored data, or loss of follow-up.

### Other outcome assessments

#### Evaluation of TCM symptoms changes

The symptoms scores are made, recorded and calculated based on graded scale of lung cancer symptoms required in "The Guiding Principles of Clinical Research of New Chinese Medicine treating Primary Bronchial Lung Cancer" (2002) issued by the State Drug Administration before and after each treatment. The total scores of single symptom are TCM symptoms scores, and symptoms efficacy assessments are based on the changes before and after treatment.

### Safety assessment

Blood routine, liver and kidney function, urination and stool routine and EKG will be measured before and after treatment to assess the toxicity and side effects of each group according to Common Terminology Criteria for Adverse Events V3.0 (CTC AE) issued by National Cancer Institute (NCI) (http://ctep.cancer.gov/).

AEs is recorded according to Common Terminology Criteria for Adverse Events V3.0 (CTC AE) made by National Cancer Institute (NCI) (http://ctep.cancer.gov/) after treatment each week.

### Adverse events (AE)

Adverse events including toxicity and side effects should be reported according to Common Terminology Criteria for Adverse Events V3.0 (CTC AE) issued by National Cancer Institute (NCI) (http://ctep.cancer.gov/). All unexpected responses possibly or clearly related to the research will be reported. If serious adverse events (SAE) occur, experimental treatments will be stopped immediately and appropriate treatments will be offered. The type and frequency of adverse events will be reported for each group.

### Statistical analysis

All the clinical data will be saved in computers after collection work and database will be set up by Microsoft Access 2007 software. Statistical analysis will be conducted on an intention-to-treat basis with a 95% confidence interval using the SPSS 16.0 statistical package. For analysis of baseline characteristics, either two-sample t tests or Wilcoxon rank sum tests for continuous data and Chi-squared tests or Fisher’s exact tests for categorical data will be conducted after the test for normality. Kaplan-Meier survival analyses should be used for calculating median survival time (MST), Long-Rank is used between two groups. A significance level of 5% will be used throughout the analysis. Experts of Shanghai Medical Clinical Research Centre will be invited to manage the statistic analysis.

## Discussion

The aim of this study is to prove that whether chemotherapy combined with TCM treatment can improve patients' quality of life and prolong survival. We have presented the design and protocol for a placebo-controlled, double-blinded RCT that will provide evidence for the effectiveness of chemotherapy combined with or without TCM in QOL of postoperative NSCLC patients. The result will provide support for integrative optimization of the treatment of lung cancer patients.

## Competing interests

All the authors have no conflicts of interest.

## Authors’ contribution

LX, JXL, HGL, ZYX and LJJ were responsible for identifying the research question, designing of the study, obtaining ethics approval, the acquisition of funding and overseeing study implementation. All authors were responsible for the manuscript drafting and have read and approved the final version.

## Pre-publication history

The pre-publication history for this paper can be accessed here:

http://www.biomedcentral.com/1472-6882/12/112/prepub
